# Patient satisfaction and health services in two public hospitals in Mogadishu, Somalia: a cross-sectional study

**DOI:** 10.3389/frhs.2025.1552322

**Published:** 2025-07-04

**Authors:** Abdifatah Abdullahi Jalei, Yahye Sheikh Abdulle Hassan, Abdifetah Ibrahim Omar, Mohamed Abdi Ali

**Affiliations:** ^1^ Faculty of Medicine and Health Sciences, Jamhuriya University of Science and Technology, Mogadishu, Somalia; ^2^ Jamhuriya Research Center, Jamhuriya University of Science and Technology, Mogadishu, Somalia

**Keywords:** patient satisfaction, healthcare services, humanness of care, service quality, Somalia

## Abstract

**Introduction:**

Healthcare system performance widely recognizes patient satisfaction as a pivotal measure that directly influences both the quality of care and health outcomes. It reflects patients’ experiences and perceptions of healthcare delivery, which are essential for identifying gaps and implementing improvements in health services. In conflict-affected regions like Somalia, understanding the factors that shape patient satisfaction is crucial for optimizing healthcare delivery and building trust between healthcare providers and patients. This study investigates patient satisfaction and its provider-related determinants at Banadir and De Martino hospitals in Mogadishu, Somalia.

**Methods:**

A cross-sectional study was conducted from May to August 2023, involving 384 adult patients (aged 18 years and older) seeking outpatient healthcare services at two public hospitals in Mogadishu, Somalia. Data were collected using structured questionnaires and analyzed using STATA 16 software.

**Results:**

Of the 384 participants, 58.6% were under 30 years of age, and 88% were female. The overall patient satisfaction rate was 53.13%. While 61.98% of patients were unsatisfied with accessibility, but 73.70% were satisfied with the humanness of care. Most participants were married (68.2%) and illiterate (62.2%). Significant associations were found between patient satisfaction and education level (*p* = 0.009), as well as income (*p* < 0.001). Other demographic factors did not significantly influence satisfaction levels.

**Discussions:**

The study found a modest patient satisfaction with public hospitals in Mogadishu. The lowest domain score was for continuity of care, while the highest was for humanness of care. These findings provide crucial baseline data for enhancing service quality and patient-centered care in Somalia's conflict-affected urban healthcare settings.

## 1 Introduction

Patient satisfaction refers to the degree to which patients feel their expectations have been met after receiving healthcare services, serving as a critical indicator of quality and reflecting the gap between anticipated and actual services (1). It provides invaluable insights into patient needs, guiding healthcare improvements and influencing treatment adherence and continued healthcare engagement (2, 3). Patient satisfaction has become an important component in the evaluation of healthcare services globally, such as in quality assurance and accreditation process (1, 4). The public health sectors in resource-limited settings, such as Somalia, often face challenges, including underfunding, inadequate infrastructure, and shortage of skilled workers, which can directly impact patient satisfaction. In such settings, patient satisfaction plays a crucial role not only in health service utilization but also in compliance with treatment regimens (5). Patient satisfaction is influenced by various factors, including communication, administrative functions, and service quality. Several studies underscore the importance of effective communication between healthcare providers and patients as key determinants of patient satisfaction (6, 7). For instance, a study in rural China revealed that improving communication skills among healthcare providers significantly enhanced patient satisfaction (8). Similarly, in Malaysia, factors such as interpersonal conduct and effective communication were valued by patients, further reinforcing the role of these aspects in shaping patient satisfaction levels (9).

Recent advancements in healthcare service delivery including AI-based patient satisfaction prediction models, evaluation methodologies that emphasize adaptive feedback loops, and engineering frameworks in low-income countries (10, 11). Incorporating such interdisciplinary tools offers valuable directions for enhancing healthcare quality, especially in fragile contexts like Somalia.

In Somalia, the healthcare system remains fragile, emerging slowly from decades of internal-conflicts and civil war. The majority of healthcare services are provided by private hospitals, with limited government regulation and oversight. These facilities often face critical challenges, including limited resources, overcrowded conditions, and insufficient regulatory frameworks, all of which can significantly impact patient satisfaction (12). Despite these challenges, significant steps must be taken to address these deficiencies and enhance patient satisfaction. Evidence from other low-income countries suggests that provider-related factors, such as communication skills, competence, and interpersonal care, are key drivers of patient satisfaction (13). However, little is known about how these factors influence patient satisfaction within the Somali healthcare context, leaving a critical gap in understanding.

This study seeks to fill this gap by assessing patient satisfaction and provider-related determinants in two major public hospitals, Banadir and Demartino, in Mogadishu, Somalia. By examining patient perspectives, this research aims to identify critical areas for improvement, ultimately contributing to better healthcare service delivery.

## 2 Materials and methods

### 2.1 Study design and period

A cross-sectional study was carried out from May to August 2023 in Banadir and De Martino hospitals, the largest public hospitals under the federal government of Somalia.

### 2.2 Study population

The study population was adult patients (18 years and older) who received medical care in the outpatient department of two state-run hospitals Mogadishu, Somalia.

### 2.3 Inclusion and exclusion criteria

All patients who visited public hospitals during the data collection period were included in the study. Seriously ill patients and those with mental illness were excluded from the study. Additionally, patients who failed to provide consent were also excluded.

### 2.4 Sample size determination

The sample size was determined using a single population proportion formula for cross-sectional studies. The formula utilized was:

$$n=\frac{\frac{Za}{2}pq}{d^{2}}$$

Where Z is the critical value at a 95% confidence interval, *P* is assumed to be 50% since the proportion of patient satisfaction is unknown, and *d* is the margin of error at 0.05. Consequently, the final sample size was 384.

### 2.5 Research instrument

#### 2.5.1 Data collection

A questionnaire developed for this study was used as a data collection tool. Two experts assessed its content validity. The questionnaire was initially developed in English and translated into the Somali language. A pilot test was conducted on a sample of 30 participants to assess internal consistency, yielding a satisfactory Cronbach's alpha of 0.82. The questionnaire comprises two sections. The first section collected sociodemographic information, including gender, age, educational background, employment status, and monthly income range. The second section, labelled “Satisfaction Measurement,” assessed patient satisfaction with hospital services and contained five domains. These dimensions were intended to cover aspects such as accessibility of health services (5 questions), continuity of healthcare services (4 questions), humanness (6 questions), comprehensiveness of healthcare services (6 questions), and communication of healthcare services (4 questions).

### 2.6 Data analysis

After the principal investigator inspected the data for completeness, it was entered into Excel and then transferred into STATA version 16.1. Descriptive statistics were used to analyze frequency, percentage, mean and standard deviation of variables. Once normality was verified, the mean score was used to determine overall patient satisfaction. Patients scoring below the mean (18.23) were categorized as dissatisfied, while those scoring at or above the mean were categorized as satisfied. The chi-square test was used to assess the association between the outcome variable (patient satisfaction) and sociodemographic variables. *P*-value less than 0.05 was considered statistically significant.

### 2.7 Ethical considerations

The study obtained ethical approval from Jamhuriya University of Science Research Ethics Committee (Ref: VPR&D/1008/EC/052023). Written informed consent was obtained from all participants before their involvement in the study.

## 3 Results

### 3.1 Sociodemographic characteristics of participants

The study sample consisted of 384 respondents. As shown in Table 1, a significant majority of participants (58.6%) were aged below 30 years, with females comprising the majority. Among the respondents, 68.2% were married, and 62.2% were illiterate. Furthermore, most participants (77.1%) were unemployed.

**Table 1:** Demographic characteristics for the study participants.

Variable	Freq.	Percent (%)
Age (years)		
<30	225	58.6
>31	159	41.4
Sex		
Male	46	12.0
Female	338	88.0
Marital status		
Single	52	13.5
Married	262	68.2
Divorced	50	13.0
Widowed	20	5.2
Educational level		
Illiterate	239	62.2
Primary	77	20.1
Secondary	43	11.2
University	25	6.5
Occupation		
Employed	88	22.9
Unemployed	296	77.1
Monthly income		
<100 $	298	77.6
101–200 $	66	17.2
>201 $	20	5.2

### 3.2 Patient satisfaction with various aspects of healthcare services

A significant proportion of the participants (61.98%) in the study expressed dissatisfaction with the accessibility of healthcare services. Conversely, a significant majority (55.73%) were satisfied with the continuity of healthcare services. Regarding the humanness of healthcare services, a substantial majority (73.70%) reported satisfaction. Regarding the comprehensiveness, a significant majority (62.24%) indicated they were satisfied. However, a notable proportion (54.95%) expressed dissatisfaction with the communication of healthcare services. Overall, the study found that 53.13% of participants reported satisfaction with the healthcare services they received (Table 2).

**Table 2:** Patient satisfaction with healthcare services.

Accessibility of health service	Freq.	Percent (%)
Dissatisfied	238	61.98
Satisfied	146	38.02
Continuity of healthcare service		
Dissatisfied	170	44.27
Satisfied	214	55.73
Humanness of care		
Dissatisfied	101	26.30
Satisfied	283	73.70
Comprehensiveness of healthcare services		
Dissatisfied	145	37.76
Satisfied	239	62.24
Communication of healthcare services		
Dissatisfied	211	54.95
Satisfied	173	45.05
Overall satisfaction		
Dissatisfied	180	46.88
Satisfied	204	53.13

### 3.3 Mean scores of patient satisfaction across different healthcare dimensions

The overall mean satisfaction score on a five-point scale was 3.65 (95% CI: 3.56–3.73) as shown in Table 3. The highest satisfaction was reported for the humanness of care, with a mean score of 4.93 (95% CI: 4.80–5.06). In contrast, the lowest satisfaction scores were observed for continuity of healthcare services and accessibility, with mean scores of 2.62 (95% CI: 2.51–2.73) and 3.02 (95% CI: 2.89–3.15), respectively.

**Table 3:** Mean of patient satisfaction for the five studied dimensions.

Variable	Mean	SD	SE	95% CI
Accessibility of health service	3.02	1.32	0.07	2.89–3.15
Continuity of healthcare service	2.62	1.10	0.06	2.51–2.73
Humanness	4.93	1.31	0.07	4.80–5.06
Comprehensiveness of Healthcare Services	4.49	1.48	0.08	4.34–4.64
Communication of healthcare services	3.17	0.92	0.05	3.08–3.27
Overall satisfaction	3.65	1.23	0.07	3.56–3.73

SD, standard deviation; SE, standard error; CI, confidence interval.

### 3.4 Association between sociodemographic characteristics of patient and their satisfaction

The study examined the relationship between patients’ satisfaction and various demographic and socioeconomic factors as shown in Table 4. No statistically significant associations were found between patient satisfaction and sex (*χ*² = 0.0314, df = 1, *p* = 0.859), age (*χ*² = 0.2762, df = 1, *p* = 0.599), marital status (*χ*² = 3.2127, df = 3, *p* = 0.360), or occupation (*χ*² = 1.9573, df = 1, *p* = 0.162). However, significant associations were observed for education (*χ*² = 11.6003, df = 3, *p* = 0.009) and monthly income (*χ*² = 17.1259, df = 2, *p* < 0.001). These findings indicate that educational level and income levels are associated with patient satisfaction, while the other demographic factors examined in this study do not demonstrate a significant influence on satisfaction levels.

**Table 4:** Association between patient satisfaction and sociodemographic factors.

Variable	Satisfied, *N* (%)	Dissatisfied, *N* (%)	*χ* ^2^	*P* value
Sex				
Male	25 (54.3)	21 (45.7)	0.031	0.859
Female	279 (82.5)	59 (17.5)		
Age (years)				
<30	117 (52.0)	108 (48.0)	0.276	0.599
>31	87 (54.7)	72 (45.3)		
Marital status				
Single	26 (50.0)	26 (50.0	3.213	0.360
Married	144 (55.0)	118 (45.0)		
Divorced	27 (45.0)	23 (55.0)		
Widowed	7 (35.0)	13 (75.0)		
Education				
Illiterate	134 (56.1)	105 (43.9)	11.60	0.009
Primary	45 (58.4)	32 (41.6)		
Secondary	19 (44.2)	24 (55.8)		
University	6 (24.0)	19 (76.0)		
Monthly income				
<100 $	174 (58.4)	124 (43.6)	17.126	<0.001
101–200 $	26 (36.4)	40 (63.6)		
>201 $	4 (20.0)	16 (60.0)		
Occupation				
Employed	41 (55.9)	47 (44.1)	1.957	0.162
Unemployed	163 (55.1)	133 (44.9)		

## 4 Discussions

Patient satisfaction with the quality of healthcare services is a critical factor influencing healthcare outcomes and service utilization (14). Understanding the factors associated with patient satisfaction can help improve the quality of care. Thus, this study aimed to assess patient satisfaction in two public hospitals in Mogadishu, Somalia, and examine the relationship between satisfaction levels and socio-demographic factors.

Overall, patient satisfaction in this study was 53.13%, similar to finding by Holikatti *et al*. (15) (55.3%) and Abbasi-Moghaddam *et al*. (16) in Tehran (57.5%), but lower than those reported in study from Deva *et al*. (17) in Kashmir 80%, Kumari *et al*. (18) in Lucknow 81.6%. Satisfaction levels are often linked to patients’ expectations, which tend to be higher among individuals with limited healthcare knowledge, resulting in greater satisfaction (19). The overall mean satisfaction score across the five domains was 3.65 (95% CI: 3.52, 3.76), aligning with studies from Iran with quality mean score was 3.73 (20) but lower than in Nigeria, where the mean score was 4.2 (21). The highest score was in the humanness domain (4.93 ± 1.31), indicating that the respectful behavior of healthcare staff-receptionist, nursing, lab staffs and doctors, significantly enhanced patient satisfaction. This highlights the importance of empathy in patient care (22). In contrast, continuity of care scored the lowest, likely due to limited operational hours, appointment availability, and financial barriers, which impede follow-up care.

Comprehensiveness of healthcare services was the second determinant of patient satisfaction with a mean score of 4.49. This can be attributed either to the excellent comprehensiveness of healthcare at the studied center or to patients who were not aware of medicine and medical procedures and consequently gave higher scores to this dimension. On contrary poor communication with physicians, lack of empathy, and the chronicity of many of the disorders lead to dissatisfaction (23). The mean score of continuity of healthcare services were the lowest among all the domains of patient satisfaction. The most plausible explanation could be operational challenges, such as limited operating hours and appointment slots at healthcare facilities, making it difficult for patients to schedule consistent follow-up visits. A shortage of healthcare providers leads to longer waiting times and fewer available appointments, further disrupting continuity of care. Additionally, financial barriers, such as lost wages due to time off work for appointments, can also be a significant burden.

Regarding sociodemographic factors, education level and income were significantly associated with satisfaction with healthcare center services. In contrary the pattern of satisfaction in different domains according to sociodemographic variables does not show any significant difference in a study conducted in Port Blair, India by Gaur et al. (5). However, other studies done by Young *et al*. (24) in USA and Dullie *et al*. (25) in Malawi found that demographic characteristics like age, race, and health status had a statistically significant effect on satisfaction scores. Illiterate patients were more likely to be satisfied, consistent with studies from Kuwait (26) and Ethiopian (1). In Bangladesh, higher level of education is associated with lower level of patient satisfaction (7). This shows that patients with relatively higher educational status have a greater expectation; educated individuals are more likely prone to feel small faults in the different department of health institutions like delay, extended waiting time, and lack of prescribed drugs and they are more critical in analyzing the services provided; these could make them less satisfied. Patients with lower incomes also reported higher satisfaction, as their expectations tend to be lower, as noted in other studies (27, 28). In contrast, those with higher incomes and education had greater expectations and were more critical of service deficiencies, leading to lower satisfaction. The high proportion of females (88%) in this study reflects the cultural and caregiving roles in Somali society, where women often attend healthcare facilities on behalf of their families.

A limitation of this study is the lack of comparable research in Somalia, making it challenging to benchmark the results. Additionally, patient satisfaction is subjective and influenced by personal expectations and experiences. Future research should include qualitative methods and explore healthcare workers’ perspectives to provide a more comprehensive understanding.

## 5 Conclusion

This study found that overall patient satisfaction with public hospital services in Mogadishu was 53.13%. Among the various socio-demographic variables studied, only the education level of the participants and their monthly income were significant as the predictors of patient satisfaction with hospital healthcare. Of the five-domains studied, the dimensions of humaneness received the highest satisfaction scores, while continuity healthcare services received the lowest satisfaction scores. The study findings will serve as baseline data for improving the quality of healthcare services and making them more client centered.

## Data Availability

The original contributions presented in the study are included in the article/Supplementary Material, further inquiries can be directed to the corresponding author.
